# Association of the maternal microbiome in Japanese pregnant women with the cumulative prevalence of dermatitis in early infancy: A pilot study from the Chiba study of Mother and Child Health birth cohort

**DOI:** 10.1016/j.waojou.2019.100065

**Published:** 2019-11-01

**Authors:** Hiromi Tanabe, Kenichi Sakurai, Tamotsu Kato, Yohei Kawasaki, Taiji Nakano, Fumiya Yamaide, Naoko Taguchi-Atarashi, Masahiro Watanabe, Shingo Ochiai, Hiroshi Ohno, Hideoki Fukuoka, Naoki Shimojo, Chisato Mori

**Affiliations:** a Center for Preventive Medical Sciences, Chiba University, 1-8-1 Inohana, Chuo-ku, Chiba, 260-8670, Japan; b Laboratory for Intestinal Ecosystem, RIKEN Center for Integrative Medical Sciences, 1-7-22 Suehiro-cho, Tsurumi-ku, Yokohama, Kanagawa, 230-0045, Japan; c Immunobiology Laboratory, Graduate School of Medical Life Science, Yokohama City University, 1-7-29 Suehiro-cho, Tsurumi-ku, Yokohama, Kanagawa, 230-0045, Japan; d Biostatistics Section, Clinical Research Center, Chiba University Hospital, 1-8-1 Inohana, Chuo-ku, Chiba, 260-8670, Japan; e Department of Pediatrics, Graduate School of Medicine, Chiba University, 1-8-1 Inohana, Chuo-ku, Chiba, 260-8670, Japan; f Department of Bioenvironmental Medicine, Graduate School of Medicine, Chiba University, 1-8-1 Inohana, Chuo-ku, Chiba, 260-8670, Japan; g Intestinal Microbiota Project, Kanagawa Institute of Industrial Science and Technology, 3-2-1 Sakado, Takatsu-ku, Kawasaki, Kanagawa, 213-0012, Japan; h Research Organization for Nano-Life Innovation, Waseda University, 413 Building 120-5, Waseda University Research Center, 513 Waseda-tsurumaki-cho, Shinjuku-ku, Tokyo 162-0041, Japan

**Keywords:** Birth cohort, Dermatitis in early infancy, Prenatal gut microbiota, Proteobacteria, Actinobacteria, AD, atopic dermatitis, BDHQ, Brief-Type Self-Administered Diet History Questionnaire, CB, umbilical cord blood, CP, cumulative prevalence, DEI, dermatitis in early infancy, FA, food allergy, SDI, Shannon diversity indices, TARC, thymus and activation regulated chemokine, Th1, type 1 helper T cell, Th2, type 2 helper T cell, Treg cell, regulatory T cell

## Abstract

**Background:**

The prenatal maternal microbiome, including the gut microbiota, has been suggested to influence the incidence of allergies in offspring. Moreover, epidermal barrier dysfunction in early infancy has been attributed to the development of subsequent allergies. We hypothesized that the prenatal microbiome may affect the gut microbiota, acting as an initial trigger to alter immune development in the foetus. The maternal microbial composition may be linked to the prevalence of dermatitis in early infancy (DEI) of the offspring, leading to subsequent allergic symptoms.

**Methods:**

This study was conducted as part of the Chiba Study of Mother and Child Health (C-MACH) birth cohort that was initiated in 2013; 434 healthy pregnant women at < 13 weeks of gestation were recruited. DEI was assessed for up to 4 months after birth, and allergic symptoms were determined in 10-month-old infants using questionnaires. Other information related to the maternal microbiome was obtained from questionnaires filled out during pregnancy. Stool samples were collected from pregnant women at 12 (*n* = 59) and 32 weeks (*n* = 58) of gestation, which were used for gut microbiota analysis using barcoded 16S rRNA gene sequencing.

**Results:**

Symptoms of allergy, especially of inherited allergies, show a higher prevalence at 10 months after birth in the DEI group. DEI occurrence was negatively correlated with family size and cat ownership. The diversity of Proteobacteria at 12 weeks of gestation and the relative abundance of Actinobacteria at 32 weeks of gestation in maternal feces were lower at both time points of gestation in the DEI group. In addition, the diversity of Proteobacteria in prenatal feces was negatively correlated with family size at 12 weeks, and with dog ownership at both gestational time points.

**Conclusions:**

The composition of the maternal microbiome may influence the risk of allergies in offspring, even before birth. Furthermore, the diversity of Proteobacteria and the relative abundance of Actinobacteria in maternal feces were negatively associated with DEI, which may be associated with the risk of allergy development in infancy. This early trigger may be a good predictor of allergy development during infancy and childhood.

## Background

Dermatitis, which includes infantile eczema, neonatal acne, seborrheic dermatitis, and intertrigo, is commonly encountered in neonates, and epidermal barrier dysfunction in infancy has been shown to be a primary causative mechanism of subsequent allergic symptoms.^1^ Thus, determining the presence or absence of dermatitis in early infancy (DEI) can be an effective method to predict the risk of subsequent allergies. The so-called “allergic march” represents the natural history of atopic manifestations, characterized by a typical sequence of progression of allergic symptoms in infancy from atopic dermatitis (AD) and food allergy (FA) to asthma and rhinitis.^2^ Recent epidemiological studies suggest that exposure to food antigens through the skin contributes to allergic sensitization.^3^ Children with eczema and epidermal barrier dysfunction have a higher risk of developing FA.^4^, ^5^ Moreover, these children tend to have immune responses skewed towards type 2 T helper cells (Th2).^6^ Thus, DEI can represent an early manifestation of allergies in infancy, presumably owing to an immunological alteration that occurs before birth,^7^ leading to subsequent AD and wheezing^7^ and proceeding along the allergic march throughout development.^8^

The increasing prevalence of allergies worldwide over the past few decades has been proposed to be related to a decrease in microbial exposure during pregnancy owing to several lifestyle and environmental changes, including reduced exposure to livestock and/or the increased use of antibiotics.^9^ Indeed, microbial exposure in early life, notably during the prenatal period, appears to be especially important to reduce the risk of asthma and allergic disease.^10^, ^11^ A balanced and diverse human microbial environment in early life is essential for proper immune development, both in terms of protective anti-microbial and regulatory immune responses to environmental antigens as well as regulating the response to prevent excessive reactions as manifested by atopy and asthma. Exposure to the maternal microbiota during pregnancy was discovered to exert distinct effects on regulatory T (Treg) cells, the type 1 T helper (Th1)/Th2 cell ratio, and FOXP3 demethylation in offspring,^12^ as well as influencing the expression of innate immune receptors at birth.^13^

The maternal gut microbiota during pregnancy may also play a key role in mediating the susceptibility of allergic disease in the offspring. The primary source of commensal microbiota in the infant gut is the maternal vaginal microbiota and, to a lesser extent, the gut microbiota, which the infant comes into contact with during vaginal delivery.^14^ Variations in the composition of the microbiota have been observed in allergic infants,^15^ and a high intestinal microbial diversity during the first month of life has been reported to be a more important factor to decrease the risk of subsequent AD than the prevalence of specific bacterial taxa.^16^, ^17^ The short-chain fatty acids produced by the activities of the commensal microbiota, especially butyrate, have been shown to induce the differentiation of colonic Treg cells.^18^, ^19^ Moreover, the maternal microbiome may directly influence the developing fetal immune system.^20^ Indeed, researchers have hypothesized that during pregnancy, microbial DNA and/or cell wall components are transported to the placenta and amniotic fluid, where the molecules contribute to the development of the fetal immune system.^21^ However, the specific effect of the maternal gut microbiota on the offspring's susceptibility to allergic disease has only been investigated to-date in intervention studies, such as those assessing probiotic and dietary supplementation.^22^ Importantly, no human studies have found an association between the composition of the maternal microbiome during pregnancy and the offspring's risk of allergic disease.^20^

Thus, we hypothesized that the prenatal microbiome may affect the gut microbiota and alter the immune development of the fetus, which may trigger the development of DEI and subsequent allergic symptoms. To test this hypothesis, we analyzed the association between the maternal microbiome, with a focus on the gut microbiota, and the cumulative prevalence of DEI (CP-DEI), using data from the Chiba study of Mother and Child Health (C-MACH) birth cohort.^23^

## Methods

### Study design

Given that the prevention of DEI may be an effective strategy for decreasing the risk of subsequent allergies, this study focused on the onset of DEI. This was a case cohort study (case: DEI positive; control: DEI negative) conducted as a part of the C-MACH, a birth cohort study initiated in 2013 in the Chiba and Saitama Prefectures near Tokyo, Japan. Details of the enrollment procedure have been previously described.^23^ In brief, 434 healthy pregnant women at < 13 weeks of gestation were recruited to the C-MACH to investigate the genetic and environmental factors associated with child health using multiomics strategies. During the course of the study, 68 participants withdrew their consent, resulting in a final cohort of 366 mothers.

The study protocol was approved by the ethical boards of the Biomedical Research Ethics Committee of the Graduate School of Medicine, Chiba University (Updated ID 989: application date September 20, 2019), and written informed consent was obtained from all participants of this study.

### Questionnaires and medical records

Given that fetal immune system development begins during the first trimester,^24^ we analyzed the effect of environmental factors at both 12 (first trimester) and 32 weeks (third trimester) of gestation. Self-reported questionnaires were completed at 12 and 32 weeks of gestation, and then at 1, 4, and 10 months after birth by each mother. The progravid body mass index (BMI) was calculated by measuring the height and weight before gestation based on answers in the questionnaires at 12 weeks of gestation; information on gestational age, maternal allergies, and family size was also obtained from the questionnaires at 12 weeks. Information regarding pet ownership and antibiotic use during pregnancy was obtained from the questionnaires at both 12 and 32 weeks.

The participants also answered the self-administered Brief-type Diet History Questionnaire (BDHQ)^25^ at 12 and 32 weeks of gestation. Given that the microbiome composition is affected by dietary fat and fiber contents,^26^ the information on total fiber and total fat intake was obtained from the BDHQs at both 12 and 32 weeks of gestation. Furthermore, researchers have demonstrated that there is likely a benefit to using probiotics during pregnancy resulting primarily in the prevention of eczema.^27^ Thus, fermented food intake, measured as the sum of pickle, natto, soy sauce, and miso-paste intake, was also evaluated from the BDHQs at both 12 and 32 weeks of gestation, as fermented foods frequently contain probiotic strains.

Information about the sex, delivery mode, birth height, birth weight, head circumference, birth month, and gestational age were obtained from the medical records at birth.

Among the 366 participants, some were removed from the final analysis owing to missing maternal or infant information. These numbers varied across time points and questions; therefore, the sample size used for each analysis is indicated in the respective table columns.

### Determination of atopic manifestations

Allergic mothers were defined as those who answered “yes” to the question: “Have you ever been diagnosed with allergy disease by a physician?” at 12 weeks of gestation. CP-DEI was defined when the answer was “yes” to the question: “Has your baby had bumps on the face, head, around the ears, and neck in the last four months (rash)?” at 4 months after birth. Given that the focus of this study was cutaneous barrier dysfunction in early infancy, DEI included multiple skin disorders encountered during early infancy, such as infantile eczema, neonatal acne, seborrheic dermatitis, and intertrigo.

FA was defined when the answer was “yes” or “suspected” to the question: “Has your baby been diagnosed with a food allergy by a physician?” at 10 months after birth. Wheezing was considered to be present when the answer was “yes” to the question: “Has your baby made wheezing sounds while breathing?” at 10 months and it occurred with symptoms at least 3 times.

AD was defined according to a modified International Study of Asthma and Allergies in Childhood (ISAAC) questionnaire.^28^ The ISAAC questionnaire was designed to estimate allergies in children between the ages of 13-14 and 6-7 years; therefore, we modified the questionnaire for infants according to the Japanese guidelines for AD.^29^ Specifically, if the answer was “yes” to the question: “Currently, does your baby have eczema, accompanied by itching, that keeps appearing and vanishing and has persisted for more than 2 months?” at 10 months, then AD was considered. In this case, “itching” refers to the baby's itching behavior, for instance, scraping the head. AD was only confirmed when an itchy rash detected at any time affected specific sites according to positive responses to the following question: “Where is the itching site on your baby? Hollow of the elbow, hollow of the knee, around the ankle, around the neck, around the eyes, around the ears, cheek, forearm, or lateral side of the lower limbs”.

### Measurement of umbilical cord blood thymus and activation-regulated chemokines

Umbilical cord blood (CB) samples were collected from all participants who delivered in the 3 hospitals participating in the C-MACH. The serum was separated from each blood sample by centrifugation within 24 hours of sample collection, and stored at −80 °C until analysis. The TARC/CCL17 levels were measured using a chemiluminescent immunoassay kit (Sysmex Corp., Kobe, Japan) according to the manufacturer's guidelines.

### Maternal stool samples

Stool samples were collected from maternal participants who agreed to the analysis of their feces in a hospital associated with the C-MACH at 12 (*n* = 59) and 32 weeks (*n* = 58) of gestation for gut microbiota analysis. The samples were stored at −80 °C until DNA extraction. Among these participants, one was excluded from the analysis because of the use of antibiotics during pregnancy. Other exclusions owing to missing data included 2 mothers from the maternal analysis of the association of gut microbiota diversity with allergies. Five infants because of missing delivery mode data and all the caesarean-delivered infants (*n* = 5) were excluded from the correlative analysis between gut microbiota diversity and relative abundance at 12 weeks of gestation and CP-DEI or AD and/or FA at 10 months post-birth. Moreover, 6 and 15 infants were excluded owing to missing information from the analysis between gut microbiota diversity and relative abundance at 12 weeks of gestation and CP-DEI and AD and/or FA at 10 months. An infant was excluded because of missing delivery mode data, and the caesarean-delivered infants (*n* = 7) were excluded from the analysis between gut microbiota diversity and relative abundance at 32 weeks of gestation and DEI or AD and/or FA at 10 months. Moreover, 3 and 14 infants were excluded from the infantile analysis at 32 weeks of gestation because of missing information on DEI and/or allergy 10 months after birth, respectively.

### DNA extraction from the stools

Fecal DNA extraction was performed according to a previous report with minor modifications.^30^ Approximately 5 g of feces was blended with 30 mL of methanol (Honeywell, Muskegon, MI, USA) and filtered with a 100-μm mesh filter to remove food residue after vigorous vortexing. The filtrate was centrifuged at 15,000×*g* for 10 min at 4 °C, and the supernatant was used for metabolomics analysis. DNA of the fecal microbiome was extracted from the pellet.

The pellets (50 mg) were suspended in 450 μL of TE10. To each suspension, 7.5 mg lysozyme was added (FUJIFILM Wako-Junyaku Co., Osaka, Japan) and incubated at 37 °C for 1 hour. Subsequently, 1100 units of purified achromopeptidase (FUJIFILM Wako-Junyaku, Co.) was added to the mixture and further incubated at 37 °C for 30 minutes. The suspension was added to 10% (wt/vol) sodium dodecyl sulphate with 0.6 mg of proteinase K (Merck KGaA, Darmstadt Germany) and incubated at 55 °C for 1 hour.^31^ After centrifugation, the bacterial DNA was extracted using a phenol:chloroform:isoamyl alcohol (25:24:1) solution (Nacalai Tesque, Kyoto Japan). The DNA was precipitated by adding ethanol and sodium acetate. RNase treatment and polyethylene glycol precipitation were performed. Finally, the DNA was cleaned using a PCR clean-up system (Promega Co., Madison, WI, USA).

### 16S rRNA gene sequencing

The V1-2 variable region (27F–338R) was sequenced on an Illumina MiSeq system. Use of this variable region for 16S rRNA gene sequencing has been previously demonstrated.^30^ The 16S rRNA V1–V2 amplicon was amplified using KAPA HiFi Hot Start Ready Mix (2 × ) (TaKaRa Bio Inc., Shiga Japan) with the following universal bacterial 16S rRNA gene primers: forward, TCGTCGGCAGCGTCAGATGTGTATAAGAGACAGAGRGTTTGATYMTGGCTCAG; and reverse, GTCTCGTGGGCTCGGAGATGTGTATAAGAGACAGATTACCGCGGCTGCTGG. The first reaction mixture contained 6 pmol of each primer, 12.5 ng of microbial DNA, 12.5 μL of 2 × KAPA HiFi Hot Start Ready Mix, and sterilized water to reach a final volume of 30 μL. The PCR conditions were as follows: 95 °C for 2 minutes, and then 20 cycles at 95 °C for 30 seconds, 55 °C for 30 seconds, and 72 °C for 1 minute, followed by 72 °C for 3 minutes. The PCR product was purified using AMPure XP (Beckman Coulter, Inc., CA, USA) and confirmed using electrophoresis on 3% (w/v) agarose gels.

Dual indices and Illumina sequencing adapters were attached to the PCR products using the Nextera XT Index Kit. After purification of the amplicon using AMPure XP beads, the samples were quantified using the Quant-iT PicoGreen dsDNA Assay Kit (Life Technologies Japan, Ltd., Tokyo Japan).

Mixed samples were prepared by pooling approximately equal amounts of PCR amplicons from each sample. The pooled library was analyzed using an Agilent High Sensitivity DNA Kit on an Agilent 2100 Bioanalyzer (Agilent Technologies, Foster City, CA, USA). Real-time quantitative PCR was performed on the pooled library using the KAPA Library Quantification Kit for Illumina following the manufacturer's protocols. Based on the quantification results, the sample library was denatured and diluted. A sample library with 20% denatured PhiX spike-in was sequenced on an Illumina MiSeq using a 500-cycle kit.

Taxonomic assignments and estimation of the relative abundance from the sequencing data were performed using the analysis pipeline of the QIIME software package.^32^ Chimera checking was performed using UCHIME.^33^ Operational taxonomic units (OTUs) were defined as 97% similarity. The OTU was assigned a taxon based on comparison with the Greengenes database using RDPclassifier.^34^, ^35^ The proportion of identified taxa in each sample was summarized and the bacterial diversity was calculated. Recent studies have revealed that the human gut microbiota primarily consists of 4 dominant phyla, namely, the Firmicutes, Bacteroidetes, Proteobacteria, and Actinobacteria, and their total proportion exceeds 95% of the population.^36^, ^37^ Thus, we focused our analysis on these four phyla.

### Statistical analysis

Data were considered statistically significant if the two-sided P-value was <0.05. All analyses were performed using R v3.4.1. (R Core Team (2017). R: A language and environment for statistical computing. R Foundation for Statistical Computing, Vienna, Austria. URL https://www.R-project.org/) and SAS for Windows v9.4 (SAS Institute Inc., Cary, NC, USA).

The changes in the prevalence of subsequent allergic symptoms with DEI were determined using Fisher's exact test. The changes in CP-DEI with antibiotic use during pregnancy, sex, delivery mode, and birth season were determined using Fisher's exact test. Variations in maternal age, progravid BMI, birth size, and gestational age with respect to CP-DEI were evaluated using the Wilcoxon rank sum test. The correlation of CP-DEI with allergy-related factors during pregnancy was assessed using logistic regression analysis. Correlation of the mother's and offspring's atopic manifestations with the Shannon diversity index (SDI) of the four phyla and one genus in the prenatal fecal microbiota was assessed using the Wilcoxon rank sum test. In addition, the relative abundance of the four phyla and one genus in the prenatal fecal microbiota compared to CP-DEI was determined using the Wilcoxon rank sum test. The correlation between the SDI and relative abundance of Proteobacteria in the prenatal fecal microbiota with allergy-related factors was evaluated using multiple regression analysis. The normality of the distribution of the dependent variables was checked using the Shapiro-Wilk normality test, and the data were converted to natural logarithms prior to multiple regression analysis if the variables were not normally distributed.

We also performed a sensitivity analysis to confirm the robustness of our results and a multiple imputation analysis. In brief, missing data were imputed using multivariate imputation by chained equations (MICEs) estimated from sequential multivariable models with conditional specifications.

## Results

### Characteristics of maternal and newborn populations

Overall, 73% of all infants had DEI by 4 months of age, which was slightly higher than the 60% reported by Shibuya et al., using the same method, in Japanese infants up to 3 months of age.^8^ Besides gestational age, there were no statistically significant differences in the characteristics of maternal and infant populations with and without DEI (Table 1). There were also no significant differences in maternal age, progravid BMI, maternal allergy, and antibiotic use during pregnancy between the mothers whose infants did and did not have DEI. With respect to the newborns’ characteristics, there were no significant differences in sex, delivery mode, birth height or weight, head circumference, and birth season between groups with and without DEI. Thus, considering these basic characteristics, no specific risk factor for DEI was identified. However, the gestational age of the DEI group was significantly higher than that of the no DEI group (Wilcoxon rank sum test, *P* = 0.03; Table 1).

**Table 1:** Demographic data (mean (95% CI)) and information of the newborns and their mothers.^a^

	DEI-positive (*n* = 240)	DEI-negative (*n* = 87)	*P* value
Maternal age (years)	32.4 (31.8–33.0) (unknown *n* = 3)	32.9 (31.9–33.9) (unknown *n* = 5)	0.40^c^
Progravid BMI	21.0 (20.7–21.3) (unknown *n* = 4)	21.5 (20.8–22.2) (unknown *n* = 5)	0.23^c^
Maternal allergy (positive/negative)	142/98 (unknown *n* = 0^d^)	51/36 (unknown *n* = 0^d^)	1.02^b^
Antibiotics use during pregnancy	4 (unknown *n* = 0^d^)	2 (unknown *n* = 0^d^)	0.66^b^
			
Sex of neonates (male/female)	128/112 (unknown *n* = 0)	43/44 (unknown *n* = 0)	0.51^b^
Delivery mode (spontaneous/caesarean)	199/26 (unknown *n* = 15)	68/12 (unknown *n* = 7)	0.43^b^
Birth height (cm)	49.6 (49.3–49.9) (unknown *n* = 2)	49.4 (49.0–49.8) (unknown *n* = 1)	0.15^c^
Birth weight (g)	3115.4 (3070.2–3160.6) (unknown *n* = 3)	3100.0 (3013.0–3187.0) (unknown *n* = 1)	0.71^c^
Head circumference (cm)	33.6 (33.4–33.8) (unknown *n* = 2)	33.5 (33.2–33.8) (unknown *n* = 1)	0.42^c^
Birth season (Sep to Feb/Mar to Aug)	187/50 (unknown *n* = 3)	64/20 (unknown *n* = 3)	0.65^b^
Gestational age (weeks)	39.2 (39.1–39.3) (unknown *n* = 4)	38.9 (38.6–39.2) (unknown *n* = 0)	**0.03**^c^

a Abbreviations: BMI, body mass index; DEI, dermatitis in early infancy.

b Calculated using Fisher's exact test.

c Calculated using Wilcoxon's rank-sum test; statistically significant (*P* ≤ 0.05) values are in bold.

d Blanks correspond to no utilization of medication

### DEI predisposes infants to allergic symptoms at 10 months

There was a higher prevalence of AD, other allergic symptoms such as FA and wheezing, and any symptom detected at 10 months after birth in the DEI group than in the no DEI group. However, significant differences were observed in AD, FA and any symptom in the offspring of allergic mothers (Table 2).

**Table 2:** Association between CP-DEI and prevalence of allergic symptoms at 10 months of age.^a^

Allergic symptoms at 10 months	DEI-positive (*n* = 201)	DEI-negative (*n* = 75)	*P*-value^b^	OR (95% CI)
*Maternal allergy positive*	*n* = 123	*n* = 48		
AD	44 (35.8%)	4 (8.3%)	**0.37e^−3^**	4.27 (1.44–17.24)
FA	24 (19.5%)	0 (0.0%)	**0.15e**^**−3**^	- (−)
Wheeze	5 (4.0%)	1 (2.1%)	1.00	1.94 (0.21–94.15)
Any allergic symptom	56 (45.5%)	5 (10.4%)	**1.21e**^**−3**^	4.35 (1.61–14.76)
				
*Maternal allergy negative*	*n* = 78	*n* = 27		
AD	19 (24.4%)	1 (3.7%)	**0.04**	6.51 (0.94–282.90)
FA	5 (6.4%)	1 (3.7%)	1.00	1.72 (0.18–84.82)
Wheeze	4 (5.1%)	1 (3.7%)	1.00	1.38 (0.13–70.66)
Any allergic symptom	25 (32.1%)	2 (7.4%)	**0.04**	4.29 (0.96–39.77)

a Abbreviations: CP-DEI, cumulative prevalence of dermatitis in early infancy; AD, atopic dermatitis; FA, food allergy.

b *P*-values were calculated using Fisher's exact test. Statistically significant (*P* ≤ 0.05) values are in bold

### CP-DEI is correlated with CB CCL17 level, family size, and cat ownership

After adjusting the model according to maternal allergy, the CB CCL17 level remained an independent factor that significantly correlated to CP-DEI, whereas family size and cat ownership during pregnancy were inversely correlated to CP-DEI (Table 3). Dog ownership, total fiber intake adjusted by total fat intake, or fermented food intake adjusted by total energy intake did not correlate with CP-DEI.

**Table 3:** Association between allergy-related factors at 12 and 32 weeks of gestation and CB CCL17 and CP-DEI.^a^

Allergy-related factors	OR (95% CI)	*P*-value
*Maternal environment (12 weeks of gestation)*		
family size (number)^b^	0.65 (0.48–0.89)	**0.01**
dog ownership^c^ (yes)	2.28 (0.68–7.67)	0.18
cat ownership^c^ (yes)	0.42 (0.18–1.00)	**0.05**
total fiber intake/total fat intake	6.28 (0.13–309.30)	0.36
fermented food^d^ intake/total energy intake (g/kcal)	3.30 (0.01–1143.04)	0.69
CB CCL17 level (100 pg/mL)	1.00 (1.00–1.00)	**0.02**
		
*Maternal environment (32 weeks of gestation)*		
family size (number)^b^	0.69 (0.52–0.92)	**0.01**
dog ownership^c^ (yes)	2.33 (0.70–7.78)	0.17
cat ownership^c^ (yes)	0.44 (0.19–1.00)	0.05
total fiber intake/total fat intake	1.06 (0.03–41.16)	0.97
fermented food^d^ intake/total energy intake (g/kcal)	3.50 (0.01–1286.43)	0.68
CB CCL17 level (100 pg/mL)	1.00 (1.00–1.00)	**0.01**

a All parameters were calculated using logistic regression analysis adjusted by maternal allergy; statistically significant (*P* ≤ 0.05) values are in bold. Sample sizes were as follows: 214 (DEI-positive) and 70 (DEI-negative) at 12 weeks of gestation, and 217 (DEI-positive) and 73 (DEI-negative) at 32 weeks of gestation. Abbreviations: CP-DEI, cumulative prevalence of dermatitis in early infancy.

b Family size was considered to be the same at 12 and 32 weeks of gestation.

c Blanks correspond to no pets.

d Fermented foods were summed as the intake of pickles, natto, soy-sauce, and miso-paste

### Overall bacterial community structure

**Fig. 1:**
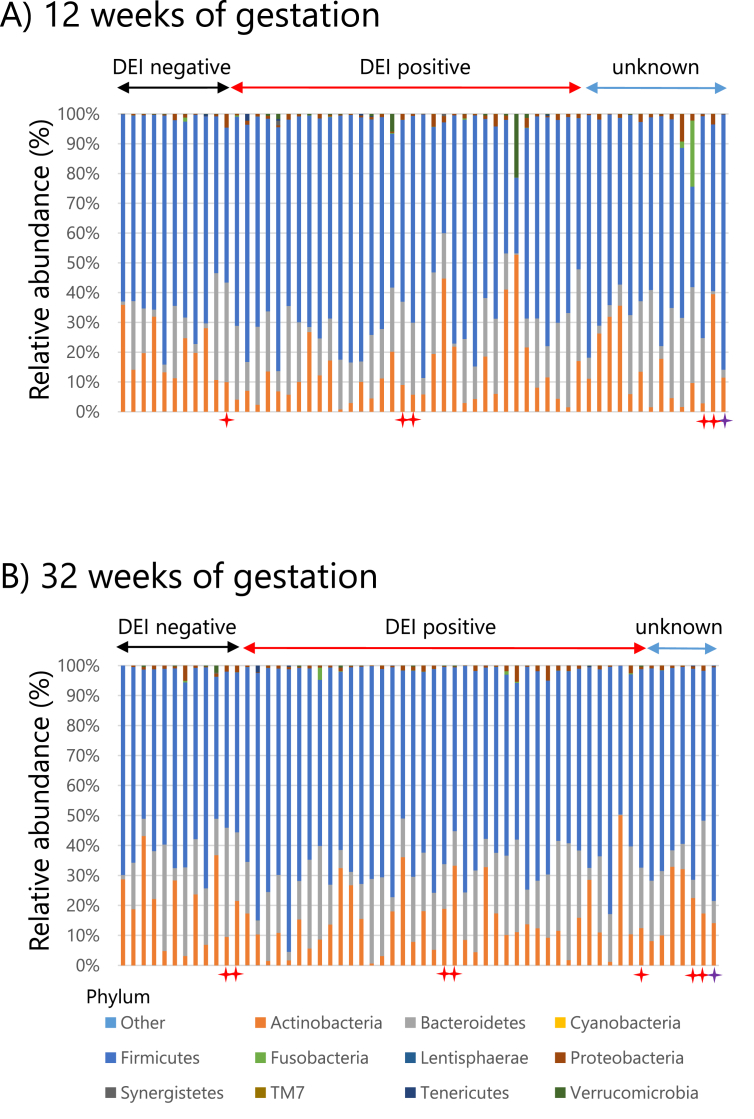
Relative abundance of bacterial communities at phylum level in maternal feces during pregnancy. Relative abundances of the 11 most abundant phyla are shown at 12 (A) and 32 (B) weeks of gestation. The remaining phyla are grouped together into “others.” The gut microbiota of mothers whose offspring were delivered by caesarean section are indicated by red stars, whereas purple stars indicate that the mother used antibiotics during pregnancy

For all fecal samples, bacterial profiles were dominated by Firmicutes (66.18% in 12 weeks; 64.71% in 32 weeks), Bacteroidetes (16.39% in 12 weeks; 17.54% in 32 weeks), Actinobacteria (14.97% in 12 weeks; 16.32% in 32 weeks), and Proteobacteria (1.27% in 12 weeks; 1.13% in 32 weeks; Fig. 1). The total abundance of the 4 dominant phyla was 98.81% and 99.8% at 12 and 32 weeks of gestation, respectively.

### Diversity of maternal fecal Proteobacteria decreased in the DEI group

For comparison of maternal fecal microbiota during pregnancy between mothers and offspring with and without atopic manifestations, we compared the SDI of four dominant phyla and one significant genus in the prenatal fecal microbiota. As shown in Table 4, the SDI of Proteobacteria decreased in the DEI group compared to the no DEI group at 12 weeks of gestation, although the difference was not statistically significant (Wilcoxon rank sum test, *P* = 0.07; Table 4-1). However, a significant decrease was observed at 32 weeks of gestation (*P* = 0.04; Table 4-2). Inversely, the total diversity of the DEI group at 12 weeks of gestation was increased compared with that of the no DEI group, although the difference was not statistically significant (*P* = 0.05; Table 4-1). No other change was observed in the prenatal fecal microbiota related to maternal allergy, or with AD and/or FA of the offspring at 10 months after birth. Missing data did not affect the sensitivity analyses.

**Table 4-1:** SDIs of 4 phyla and a significant genus in the maternal fecal microbiota at 12 weeks of gestation.^a^

12 weeks of gestation	Positive			Negative			*P* value^b^
	median	IQR	*n*	median	IQR	*n*	
*Maternal allergy*							
Total	3.73	3.44–3.90	*n* = 35	3.79	3.64–4.03	*n* = 21	0.25
Firmicutes	3.50	3.25–3.74		3.53	3.23–3.91		0.59
Proteobacteria	1.32	1.01–1.54		1.08	0.94–1.36		0.13
Actinobacteria	1.57	1.10–1.73		1.58	1.30–1.77		0.85
Bacteroidetes	1.90	1.68–2.25		2.03	1.81–2.27		0.71
*Bacteroides* sp.	1.53	1.23–1.68		1.46	1.26–1.69		0.81
*CP-DEI*							
Total	3.78	3.63–3.97	*n* = 32	3.58	3.30–3.78	*n* = 10	0.05
Firmicutes	3.59	3.28–3.84		3.41	3.22–3.64		0.14
Proteobacteria	1.14	0.94–1.42		1.50	1.21–1.60		0.07
Actinobacteria	1.59	1.29–1.73		1.65	0.97–1.75		0.94
Bacteroidetes	1.99	1.74–2.33		2.00	1.69–2.25		0.92
*Bacteroides* sp.	1.51	1.23–1.69		1.60	1.40–1.80		0.37
*AD and/or FA at 10 months*							
Total	3.65	3.40–3.86	*n* = 9	3.80	3.63–3.99	*n* = 24	0.56
Firmicutes	3.68	3.37–3.91		3.56	3.31–3.79		0.56
Proteobacteria	1.22	1.14–1.41		1.25	1.03–1.56		0.80
Actinobacteria	1.70	1.47–1.77		1.61	1.26–1.79		0.65
Bacteroidetes	1.74	1.52–2.49		2.04	1.80–2.33		0.54
*Bacteroides* sp.	1.41	1.09–1.65		1.56	1.40–1.70		0.25

a Data represent the maternal and offspring's atopic manifestations, separately. Abbreviations: SDI, Shannon diversity index; CP-DEI, cumulative prevalence of dermatitis in early infancy.

b Calculated using Wilcoxon rank sum test

**Table 4-2:** SDIs of 4 phyla and a significant genus in the maternal fecal microbiota at 32 weeks of gestation^a^

32 weeks of gestation	Positive			Negative			*P* value^b^
	median	IQR	*n*	median	IQR	*n*	
*Maternal allergy*							
Total	3.71	3.45–3.92	*n* = 35	3.64	3.53–3.98	*n* = 20	0.83
Firmicutes	3.43	3.26–3.69		3.54	3.28–3.90		0.42
Proteobacteria	1.18	1.01–1.36		1.13	0.88–1.52		0.86
Actinobacteria	1.39	1.10–1.74		1.51	1.27–1.77		0.37
Bacteroidetes	1.93	1.51–2.23		1.96	1.73–2.27		0.50
*Bacteroides* sp.	1.46	1.31–1.64		1.40	1.25–1.61		0.57
*CP-DEI*							
Total	3.68	3.45–3.91	*n* = 36	3.57	3.47–3.86	*n* = 10	0.72
Firmicutes	3.43	3.19–3.72		3.39	3.29–3.64		1.00
Proteobacteria	1.06	0.85–1.20		1.31	1.12–1.47		**0.04**
Actinobacteria	1.44	1.19–1.72		1.60	1.11–1.76		0.67
Bacteroidetes	1.89	1.51–2.22		2.05	1.71–2.32		0.54
*Bacteroides* sp.	1.45	1.25–1.64		1.52	1.32–1.74		0.63
*AD and/or FA at 10 months*							
Total	3.70	3.41–3.92	*n* = 10	3.77	3.56–3.97	*n* = 25	0.55
Firmicutes	3.48	3.36–3.81		3.58	3.33–3.89		0.63
Proteobacteria	1.06	0.72–1.24		1.15	0.98–1.32		0.40
Actinobacteria	1.43	1.27–1.65		1.52	1.20–1.79		0.44
Bacteroidetes	1.82	1.49–2.27		1.98	1.64–2.24		0.63
*Bacteroides* sp.	1.27	1.17–1.54		1.49	1.30–1.67		0.21

a Data represent the maternal and offspring's atopic manifestations, separately. Abbreviations: SDI, Shannon diversity index; CP-DEI, cumulative prevalence of dermatitis in early infancy.

b Calculated using Wilcoxon rank-sum test; statistically significant (*P* ≤ 0.05) values are in bold.

### Relative abundance of Actinobacteria and Bifidobacterium decreased in the DEI group

**Fig. 2:**
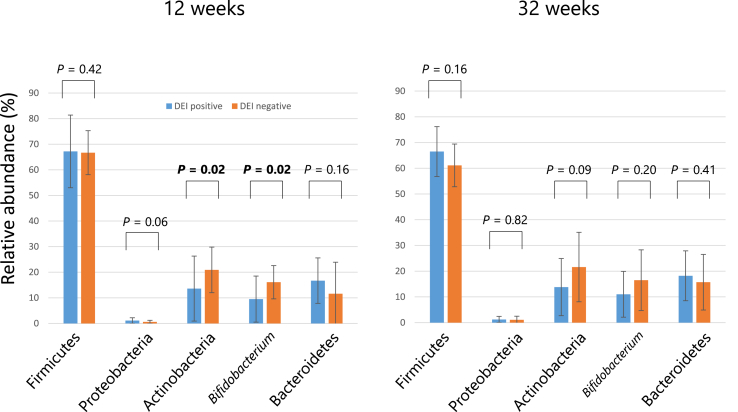
Mean relative abundance of 4 phyla and a genus in the maternal fecal microbiota. The mean relative abundance of the phyla Firmicutes, Proteobacteria, Actinobacteria, and Bacteroidetes, as well as the genus *Bifidobacterium* are shown for both the DEI positive (blue) and negative (orange) groups. *P* values were calculated using the Wilcoxon rank-sum test. Statistically significant (*P* ≤ 0.05) values are in bold

We determined the relative abundance of 4 dominant phyla and 1 significant genus in the prenatal fecal microbiota. As shown in Fig. 2, the mean relative abundance of Actinobacteria was significantly decreased in the DEI group at 12 (Wilcoxon rank sum test *P* = 0.02) and 32 weeks (*P* = 0.09) of gestation. Moreover, there was a significant decrease in the genus *Bifidobacterium* in the DEI group at 12 weeks (*P* = 0.02). In addition, the mean relative abundance of Proteobacteria increased in the DEI group at 12 weeks of gestation, although the difference was not statistically significant (*P* = 0.06). Missing data did not affect the sensitivity analyses.

### Family size and dog ownership during pregnancy is negatively associated with Proteobacteria diversity in the maternal fecal microbiota

The SDI of Proteobacteria in the maternal feces during pregnancy was negatively correlated with family size at 12 weeks and dog ownership at 12 and 32 weeks of gestation (Multiple regression analysis, *P* < 0.05; Table 5). Thus, family size and dog ownership did not contribute to the overall increase in the diversity of Proteobacteria in maternal feces. No correlation between Proteobacteria diversity and cat ownership was observed at either time point.

**Table 5:** Association between SDIs of Proteobacteria in maternal feces and maternal allergy-related environmental factors.^a^

Maternal allergic-related environmental factors	*β* value	95% CI		*P* value
		lower	upper	
*12 weeks of gestation n = 56*				
Family size (number)	−0.14	−0.26	−0.03	**0.02**
dog ownership (yes)^b^	−0.99	−1.42	−0.57	**2.1e^−5^**
cat ownership (yes)^b^	0.06	−0.52	0.64	0.83
*32 weeks of gestation n = 55*				
Family size (number)	0.02	−0.09	0.14	0.69
dog ownership (yes)^b^	−0.59	−1.03	−0.15	**0.01**
cat ownership (yes)^b^	0.25	−0.25	0.74	0.33

^a^ *β* and *P* values were calculated using multiple regression analyses; the SDIs of Proteobacteria were converted to the natural logarithm prior to multiple regression analysis. Statistically significant values are in bold (*P* ≤ 0.05). Three mothers were excluded, 1 for using antibiotics during pregnancy and 2 owing to missing family size data. Abbreviations: SDI, Shannon diversity index.

b Blanks correspond to no pets

Moreover, there was no association between the relative abundance of Proteobacteria in prenatal faeces with family size (*P* = 0.78 at 12 weeks, *P* = 0.65 at 32 weeks), dog ownership (*P* = 0.08 at 12 weeks, *P* = 0.88 at 32 weeks), or cat ownership (*P* = 0.93 at 12 weeks, *P* = 0.86 at 32 weeks). Missing data did not affect the sensitivity analyses.

## Discussion

DEI may be the initial trigger that leads to subsequent allergies in infancy, especially in the offspring of allergic mothers. In line with previous reports,^7^, ^8^ the present study found that DEI within 4 months of birth predisposed infants to not only AD but also FA by 10 months of age. A recent study further revealed that epidermal barrier dysfunction in infancy is likely a causative mechanism of subsequent allergic symptoms.^1^ DEIs are a form of epidermal barrier dysfunction with several causes, including non-allergic predisposition, and our results clearly support DEI as a strong risk factor for subsequent allergies, especially in the offspring of allergic mothers.

Our results further suggest that the diversity, but not relative abundance, of Proteobacteria species in the maternal intestine during pregnancy may play an important role in reducing the risk of DEI. We found a lower diversity of Proteobacteria at both early and late pregnancy stages in maternal feces in the DEI group compared with that of the no DEI group. Proteobacteria comprises Gram-negative bacteria and several pathogens such as those of the genera *Escherichia*, *Salmonella*, and *Vibrio*. The direct presentation of maternal intestinal microbes to the fetus may be critical for the maturation of the fetal immune system, the healthy development of which may prevent a hyper-responsive innate immune phenotype.^20^ In contrast, as abundant pathogens cause acute inflammation, the relative abundance of Proteobacteria may not be related to the prevalence of DEI.

Moreover, the abundance of the phylum Actinobacteria and of the genus *Bifidobacterium* during pregnancy appears to be associated with the prevalence of DEI. *Bifidobacterium* has been reported to be less prevalent in the stools of Japanese juveniles (aged less than 20 years) with AD compared to healthy controls.^38^ Thus, the abundant direct presentation of *Bifidobacterium* species in the maternal intestine may play an important role in the prevention of DEI.

We further show that the CB CCL17 levels were positively correlated with the prevalence of DEI, indicating that the cause of DEI in newborns was likely related to a factor occurring before birth. This is in line with the suggestion of Matsumoto et al.,^7^ who suggested that DEI development may be related to the immunological background.

The negative correlation of exposure to a large family and/or cats during pregnancy with DEI does not appear to be related to the diversity of microbes in the maternal intestine. In our study, family size and cat ownership were negatively correlated with CP-DEI, which is in line with the findings of previous studies.^13^, ^39^, ^40^ However, family size and cat ownership did not correlate with increased diversity or abundance of Proteobacteria species in maternal faeces. Thus, the mechanism by which the presence of more family members or cats prevents DEI during pregnancy likely involves factors beyond the maternal gut microbiota, such as influence on the respiratory system or maternal mental health status; however, further studies are needed to elucidate the detailed mechanisms. Moreover, it will be interesting to further explore the specific factors that contribute to the increase in the diversity of Proteobacteria species in maternal feces.

## Study limitations

It is important to note that there were some limitations to this study. This study was performed only with Japanese women, and it involved a relatively small sample size, especially for analysis of the gut microbiota, which may explain the lack of statistical rigor when accounting for multiple testing. Suitable sample size was not evaluated prior to stool collection by power analysis, as C-MACH is a pilot study. Moreover, we did not acquire data related to the gut microbiota, immune status, or sensitization towards inhalants or food among infants, which would reveal the association between the prenatal microbial environment and prevalence of allergies in the offspring. Furthermore, AD was diagnosed using a modified ISAAC questionnaire for infants, which has not been validated by other methods. Thus, this method of estimating AD prevalence is also a study limitation. Although we had information on fermented food intake, there were no specific questions related to yogurt intake, thus we could not assess the effects of yogurt on allergy development. Given that yogurt is a significant source of probiotics, including *Bifidobacterium* species, this information is important when considering the effect of fermented food intake during pregnancy has on allergies in the offspring.

## Conclusions

We demonstrate that the diversity of Proteobacteria and relative abundance of Actinobacteria were reduced in maternal feces during pregnancy in cases of DEI, and that DEI is associated with the risk of subsequent allergy development in infancy, especially in those at risk of inherited allergies. This early trigger can be a good predictor of subsequent allergies and allergic march through infancy and childhood. Moreover, we found a negative correlation between family size and cat ownership with the prevalence of DEI, suggesting that these factors could prevent the development of allergic symptoms in infancy. However, this negative correlation is not related to the diversity of microbes in the maternal intestine, suggesting an alternate mechanism that is worthy of further study. Nevertheless, this is the first study to link the maternal microbiome composition with risk of allergic disease in the offspring, even before birth.

## Availability of data and materials

The data that support the findings of this study are available from the C-MACH research committee upon reasonable request; however, restrictions apply to the availability of these data, which were used under license for the current study, and thus are not presently publicly available.

## Potential competing interests

The authors declare no competing interests.

## Financial disclosure

This research was supported by a grant from the Japan Society for the Promotion of Science (16H01781) and by the Chiba Foundation for Health Promotion & Disease Prevention.

## Authors’ contributions

H.T. conceived the study design, performed data analysis, and wrote the manuscript. K.S., N.S., T.N., and F.Y. participated in the study design and revised the manuscript. T.K., N.A., H.O., and H.T. performed the experiments, data analysis, and revised the manuscript. T.K. wrote the manuscript section on 16S rRNA sequencing. Y.K. completed the multivariate analysis and revised the manuscript. S.O. and M.W. participated in data collection and protocol development. H.F. supervised the study design and protocol development. C.M. participated in the conceptualization of the study design and revised the manuscript. K.S. and C.M are the guarantors of this work and, as such, had full access to all the data used in this study and take responsibility for the integrity of the data and the accuracy of the data analysis. All authors read and approved the final manuscript.

## References

[1] Kubo A., Nagao K., Amagai M. (2012). Epidermal barrier dysfunction and cutaneous sensitization in atopic diseases. J Clin Investig.

[2] Spergel J.M., Paller A.S. (2003). Atopic dermatitis and the atopic march. J Allergy Clin Immunol.

[3] Lack G., Fox D., Northstone K., Golding J. (2003). Factors associated with the development of peanut allergy in childhood. N Engl J Med.

[4] Kumar R., Caruso D.M., Arguelles L. (2010). Early life eczema, food introduction, and risk of food allergy in children. Pediatr Allergy Immunol Pulmonol.

[5] Lack G. (2012). Update on risk factors for food allergy. J Allergy Clin Immunol.

[6] Matsumoto K., Saito H. (2013). Epicutaneous immunity and onset of allergic diseases - per-"eczema"tous sensitization drives the allergy march. Allergol Int.

[7] Matsumoto K., Shimanouchi Y., Kawakubo K. (2005). Infantile eczema at one month of age is associated with cord blood eosinophilia and subsequent development of atopic dermatitis and wheezing illness until two years of age. Int Arch Allergy Immunol.

[8] Shibuya N., Saito E., Karasawa C. (2013). [Dermatitis in early infancy as a risk factor for sensitization and allergic diseases during the first year of life]. Arerugi.

[9] Jenmalm M.C. (2017). The mother-offspring dyad: microbial transmission, immune interactions and allergy development. J Intern Med.

[10] von Mutius E., Vercelli D. (2010). Farm living: effects on childhood asthma and allergy. Nat Rev Immunol.

[11] Ege M.J., Mayer M., Normand A.C. (2011). Exposure to environmental microorganisms and childhood asthma. N Engl J Med.

[12] Schaub B., Liu J., Hoppler S. (2009). Maternal farm exposure modulates neonatal immune mechanisms through regulatory T cells. J Allergy Clin Immunol.

[13] Roduit C., Wohlgensinger J., Frei R. (2011). Prenatal animal contact and gene expression of innate immunity receptors at birth are associated with atopic dermatitis. J Allergy Clin Immunol.

[14] Nagpal R., Tsuji H., Takahashi T. (2016). Sensitive quantitative analysis of the meconium bacterial microbiota in healthy term infants born vaginally or by cesarean section. Front Microbiol.

[15] Penders J., Stobberingh E.E., van den Brandt P.A., Thijs C. (2007). The role of the intestinal microbiota in the development of atopic disorders. Allergy.

[16] Abrahamsson T.R., Jakobsson H.E., Andersson A.F., Bjorksten B., Engstrand L., Jenmalm M.C. (2012). Low diversity of the gut microbiota in infants with atopic eczema. J Allergy Clin Immunol.

[17] Abrahamsson T.R., Jakobsson H.E., Andersson A.F., Bjorksten B., Engstrand L., Jenmalm M.C. (2014). Low gut microbiota diversity in early infancy precedes asthma at school age. Clin Exp Allergy.

[18] Furusawa Y., Obata Y., Fukuda S. (2013). Commensal microbe-derived butyrate induces the differentiation of colonic regulatory T cells. Nature.

[19] Furusawa Y., Obata Y., Hase K. (2015). Commensal microbiota regulates T cell fate decision in the gut. Semin Immunopathol.

[20] Vuillermin P.J., Macia L., Nanan R., Tang M.L., Collier F., Brix S. (2017). The maternal microbiome during pregnancy and allergic disease in the offspring. Semin Immunopathol.

[21] Abrahamsson T.R., Wu R.Y., Jenmalm M.C. (2015). Gut microbiota and allergy: the importance of the pregnancy period. Pediatr Res.

[22] Jenmalm M.C., Duchen K. (2013). Timing of allergy-preventive and immunomodulatory dietary interventions - are prenatal, perinatal or postnatal strategies optimal?. Clin Exp Allergy.

[23] Sakurai K., Miyaso H., Eguchi A. (2016). Chiba study of Mother and Children's Health (C-MACH): cohort study with omics analyses. BMJ Open.

[24] Spencer J., Dillon S.B., Isaacson P.G., MacDonald T.T. (1986). T cell subclasses in fetal human ileum. Clin Exp Immunol.

[25] Kobayashi S., Murakami K., Sasaki S. (2011). Comparison of relative validity of food group intakes estimated by comprehensive and brief-type self-administered diet history questionnaires against 16 d dietary records in Japanese adults. Public Health Nutr.

[26] Wu G.D., Chen J., Hoffmann C. (2011). Linking long-term dietary patterns with gut microbial enterotypes. Science.

[27] Fiocchi A., Pawankar R., Cuello-Garcia C. (2015). World allergy organization-McMaster university guidelines for allergic disease prevention (GLAD-P): probiotics. World Allergy Organ J.

[28] (1998). Worldwide variation in prevalence of symptoms of asthma, allergic rhinoconjunctivitis, and atopic eczema: ISAAC. The International Study of Asthma and Allergies in Childhood (ISAAC) Steering Committee. Lancet.

[29] Katayama I., Aihara M., Ohya Y. (2017). Japanese guidelines for atopic dermatitis 2017. Allergol Int.

[30] Kim S.W., Suda W., Kim S. (2013). Robustness of gut microbiota of healthy adults in response to probiotic intervention revealed by high-throughput pyrosequencing. DNA Res.

[31] Morita H., Kuwahara T., Ohshima K. (2007). An improved DNA isolation method for metagenomic analysis of the microbial flora of the human intestine. Microb Environ.

[32] Caporaso J.G., Kuczynski J., Stombaugh J. (2010). QIIME Allows Analysis of High-Throughput Community Sequencing Data Nat Methods.

[33] Edgar R.C., Haas B.J., Clemente J.C., Quince C., Knight R. (2011). UCHIME improves sensitivity and speed of chimera detection. Bioinformatics.

[34] McDonald D., Price M.N., Goodrich J. (2012). An improved Greengenes taxonomy with explicit ranks for ecological and evolutionary analyses of bacteria and archaea. ISME J.

[35] Wang Q., Garrity G.M., Tiedje J.M., Cole J.R. (2007). Naive Bayesian classifier for rapid assignment of rRNA sequences into the new bacterial taxonomy. Appl Environ Microbiol.

[36] Nishijima S., Suda W., Oshima K. (2016). The gut microbiome of healthy Japanese and its microbial and functional uniqueness. DNA Res.

[37] Tyakht A.V., Kostryukova E.S., Popenko A.S. (2013). Human gut microbiota community structures in urban and rural populations in Russia. Nat Commun.

[38] Watanabe S., Narisawa Y., Arase S. (2003). Differences in fecal microflora between patients with atopic dermatitis and healthy control subjects. J Allergy Clin Immunol.

[39] Strachan D.P. (1989). Hay fever, hygiene, and household size. BMJ.

[40] Chong S.N., Chew F.T. (2018). Epidemiology of allergic rhinitis and associated risk factors in Asia. World Allergy Organ J.

